# Public health and social measures during health emergencies such as the COVID-19 pandemic: an initial logic model to conceptualise and classify measures: Eva Rehfuess

**DOI:** 10.1093/eurpub/ckac129.534

**Published:** 2022-10-25

**Authors:** E Rehfuess, J Burns, R Ludolph, A Movsisyan, L Pfadenhauer, B Strahwald

**Affiliations:** 1 Institute for Medical Information Processing, University of Munich, Munich, Germany; 2 High Impact Events Preparedness Unit, WHO, Geneva, Switzerland

## Abstract

**Issue/problem:**

In the context of the COVID-19 pandemic, public health and social measures (PHSM) are being implemented worldwide, but in very different ways. This is also due to a lacking common understanding of PHSM so far. As a result, there are limited insights regarding their components, implementation and effectiveness as well as impacts beyond health of PHSM.

**Description of the problem:**

The project contributes to the WHO PHSM initiative. A logic model is being developed that offers a shared language and understanding of how PHSM are intended to achieve transmission-related outcomes, but also that non-intended consequences need to be considered. The overall approach is informed by a system-based logic model template and a staged approach to logic modeling. The development process is making use of (i) existing COVID PHSM taxonomies and related literature, (ii) existing theoretical frameworks related to complex interventions in complex systems, (iii) consultations with WHO staff and the according PHSM steering group, and (v) iterative brainstorming within the working group.

**Results:**

The initial logic model is rooted in a complexity perspective which recognises that (i) all measures have both intended and unintended consequences for health and society and (ii) all elements within the logic model are interconnected and interact with each other. All PHSM operate through two basic mechanisms: reducing contacts and making contacts safer. Taken together, these two mechanisms work to reduce transmission-relevant contacts. Any specific PHSM is defined by a combination of the measure itself, the population and/or setting targeted and the mode of enactment. The central hub of the logic model connects and integrates all elements. The initial logic model was applied to school and travel measures as examples.

**Main messages:**

The PHSM logic model is a conceptual basis to facilitate research on PHSM, monitoring and evaluation of PHSM, and evidence-informed decision-making.

